# A comprehensive procedure to develop water quality index: A case study to the Huong river in Thua Thien Hue province, Central Vietnam

**DOI:** 10.1371/journal.pone.0274673

**Published:** 2022-09-15

**Authors:** Hop Nguyen Van, Hung Nguyen Viet, Kien Truong Trung, Phong Nguyen Hai, Chau Nguyen Dang Giang

**Affiliations:** 1 Department of Chemistry, University of Sciences, Hue University, Hue City, Vietnam; 2 Department of Natural Resources and Environment, Thua Thien Hue province, Hue City, Vietnam; 3 Department of Natural Resources and Environment, Quang Tri province, Dong Ha City, Vietnam; The University of Auckland - City Campus: University of Auckland, NEW ZEALAND

## Abstract

This work proposed a novel procedure of Water Quality Index (WQI) development that could be used for practical applications on a local or regional scale, based on available monitoring data. Principal component analysis (PCA) was applied to the monthly data of 11 water quality parameters (pH, conductivity (EC), total suspended solid (TSS), dissolved oxygen (DO), five -day biological oxygen demand (BOD), chemical oxygen demand (COD), ammonia (N-NH_4_), nitrate (N-NO_3_), phosphate (P-PO_4_), total coliform, and total dissolved iron monitored at 11 sites at Huong river in the years 2014–2016. From the PCA, the three extracted principal components explained 67% of the total variance of original variables. From the set of communality values, the weight (w_i_) for each parameter was determined. Linear sub-index functions were established based on the permissible limits from the National Technical Regulations on Surface Water Quality set up by the Vietnam Environment Agency (VEA) to derive the sub-index (q_i_) for each parameter. The multiplicative formula that is the product of the sub-indices (q_i_) raised to the respective weights (w_i_), was used for calculation of the final WQI values. The proposed index (WQI) was then applied to the river with quarterly data of the 11 parameters monitored at ten sites in the years 2017–2020. The WQI representatively reflected the actual status of the river overall water quality, of which 97.8% of the WQI values belonged to grades of EXCELLENT and GOOD, and 2.2% of grade MODERATE. Comparison between the river water quality evaluations resulting from the developed WQI with the WQI adopted by National Sanitation Foundation (NSF-WQI) and the index issued by Vietnam Environment Agency (VN-WQI) indicated that the proposed WQI was more suitable for river quality assessment.

## Introduction

Water quality is important information in water resources management. Different uses of water need various water quality parameters consisting of physical, chemical, and biological ones. For the water quality assessment, water quality standards or guidelines have been established on international and regional scale. However, they provide evaluation taking individual parameters into account and do not indicate a general picture of the water quality in sites or regions under study [1–5]. The development of water quality assessment methods based on a quantitative and comprehensive index has attracted big concerns from scientists. Water Quality Index (WQI) is a mathematical tool to transfer water quality parameters to a single integer value, depicting the overall health status of a water body [2,6–8]. The WQI developed by Brown et al. [1] was proposed by National Sanitation Foundation (NSF-WQI) to assess surface water quality. The NSF-WQI has been applied worldwide as originally proposed or modified before applications [2,9–11]. Many reviews about developed WQIs [2,4,5,9] indicated that WQIs has been widely used as an efficient tool to assess surface and underground water quality.

According to the reviews mentioned above, the remarks were extracted as follows [4,10,12]: (i) although many WQIs are available, there is still a need for an overall WQI that can incorporate the available data and describe the water quality for different uses; (ii) significant discrepancies were observed in the course of water quality classification from different methodologies; (iii) the most challenging aspect is that WQIs are developed for a specific region, being source-specific; therefore, there is a continuing interest to develop accurate WQIs that suit a local or regional area; (iv) no single WQI has been globally accepted; (v) there is no worldwide accepted method guiding steps for WQI development, thus, further works in this fields are still necessary to solve the limitations of worldwide developed WQIs. These conclusions indicate a desire to develop a method and a water quality index for practical applications on local or regional scale, based on available monitoring data.

The aim of establishing a WQI is to transform the concentrations of selected water quality parameters (or variables) with different units and dimensions into sub-indexes with dimensionless scale, defining subindices, and choosing an aggregation method to generate the numerical value for the index [2,4,10]. The general procedure to create a WQI consists of the following steps [2,4,5]: (i) selection of water quality parameters; (ii) computation of sub-index values through a transformation of the parameters to a standard scaling factor; (iii) estimation of weights for all parameters; (iv) aggregation of the sub-index values and weights to obtain the final WQI.

### Selecting parameters

Based on a review of 30 existing WQIs, the parameters selected to calculate WQIs were divided into three types: fixed, open, and mixed systems [4]. The most of those WQIs have used a fixed set of parameters that is commonly called “basic” as the selected parameters are the most significant ones for water quality evaluation in the study site or region [1,2,12–18]. The fixed system (e.g. NSF-WQI with 9 parameters), allows users to compare water quality status among the sites or rivers, but not to add the new parameter(s) needed for assessment of water quality [19]. Some WQIs use an open system that has no guidelines for the selection of parameters, for example, the WQI developed by Canadian Council of Ministers of Environment [20]. This system causes difficulty in comparisons among monitored sites and among river basins [21]. The mixed system consists of the basic and additional parameters. The selection of additional parameters incorporated into WQI calculation is depended on their sub-index values or importance in river water quality reflection [13]. Many studies indicated that the objective (less subjective) way to select parameters for the development of a WQI is based on the results obtained from statistical analysis of available monitoring data, such as correlation analysis, multivariate analysis technique: principal component analysis/PCA, factor analysis/FA [2–4,22–24]. The issues mentioned above, relating to parameter selection for WQI development, indicate that a mixed system should be chosen to avoid ‘rigidity’ and the parameters selected should be ones monitored routinely, of great importance in reflecting river water quality.

### Defining sub-indices

This step aims to transform concentrations of selected water quality parameters into a standardized or common scale without unit, typically within identical range, i.e. 0 (poorest) - 100 (best) or 0 (poorest) - 1 (best), called sub-index [2]. To define sub-index value, WQI developers have established the sub-index functions or rating curves of different parameters [4,9]. There are three methods that are usually employed: (i) expert judgment such as the NSF-WQI [1], Oregon Index [12], and Almeida’s Index [18]; (ii) use of the water quality standards or guidelines [12–14,16,23,25–27] and (iii) statistical methods. The use of water quality standards or guidelines facilitates sub-division of sub-index values and provides more information for the users [12]. Several procedures to calculate WQI directly from the parameters without transforming them into a common scale. For instance, the CCME-WQI development process [20] uses a specific mathematic equation for directly aggregating the index.

### Estimating weights

The weights are assigned to the selected parameters concerning their relative importance and their influence on the final index value [2,4]. The weights of the parameters can be either equal or unequal. A few of WQIs used equal weights in the calculation [13,14,20,23,28–30]. Many WQIs were calculated with unequal weights. The weights assigned to the parameters were commonly defined by either participatory-based procedure such as Delphi method [1] or Analytical Hierarchy Process [31], or multivariate statistical analysis, mainly PCA and FA. To avoid subjective judgment from experts in the participatory-based procedure, the index developers suggested using PCA and FA to define parameter weights by different approaches [11,22,24,32–34]. Exploratory factor analysis (FA) is a dimension reduction method, similar in some respect to PCA, though different enough from PCA that the two should not in any real way be considered equivalent [35]. In practice, PCA is a relatively simple technique when compared to FA. With factor analysis, since there are so many options and complexities, the outcome of the procedure for any analysis may be different, depending on how many factors-remained solutions [35,36]. A big deal for FA is the non-uniqueness of loadings. This means that how well a given variable load onto a given factor often depends on how many factors were extracted in the factor analysis [35,36]. Other than FA, from PCA results, a given variable loading onto an extracted principal component is unique [35]. This means that the variable loadings obtained from PCA reflect intrinsic and actual influence or importance of the variables to the water body under study. Thus, a comprehensive and unique approach based on only PCA results to define the weights of water quality parameters is necessary for WQI development.

### Aggregating the sub-index values into final WQI

Index aggregation is conducted after the assignment of weights to obtain the final WQI value. The two most common methods to aggregate the sub-indices are the additive (arithmetic) and multiplicative (geometric) methods. There are also other modified versions of the two methods [2,4]. The mixed aggregation methods (combination of additive and geometric methods) are proposed by some researchers [16,23,30]. The multiplicative method which is shown in Eq (1) has been adopted for final aggregation in many WQIs [1,11,18,37,38].

$$\text{WQI}=\prod_{\text{i}=1}^{\text{n}}{\text{q}}_{\text{i}}^{{\text{w}}_{\text{i}}}$$ Eq (1)

Where n is number of selected parameters for WQI calculation; q_i_ and w_i_ is sub-index and weight of the *i* parameter, respectively.

The aggregation method to create the final WQI value must be selected so that it avoids problems of eclipsing and ambiguity [2]. The eclipsing arises wherein the final index value does not represent the actual state of overall water quality as the lower values of one or some sub-indices are dominated by the higher values of other sub-indices or vice versa. The ambiguity occurs wherein actual water quality is good, but final WQI answers to be bad or vice versa [4,17,19,39,40].

With the aim at developing a comprehensive and simple WQI procedure, using available monitoring data, this study is based on the following approaches: (i) a mixed system is used in parameter selection (basic and additional parameters); (ii) PCA is applied to estimate relative weights of parameters; (iii) Sub-indices are determined based on linear equations that are derived from national water quality guidelines; (iv) multiplicative formula is used as an aggregation method to calculate final WQI. This WQI procedure then is applied to Huong river in Thua Thien Hue province, Central Vietnam.

## Materials and methods

### Study area

**Fig 1:**
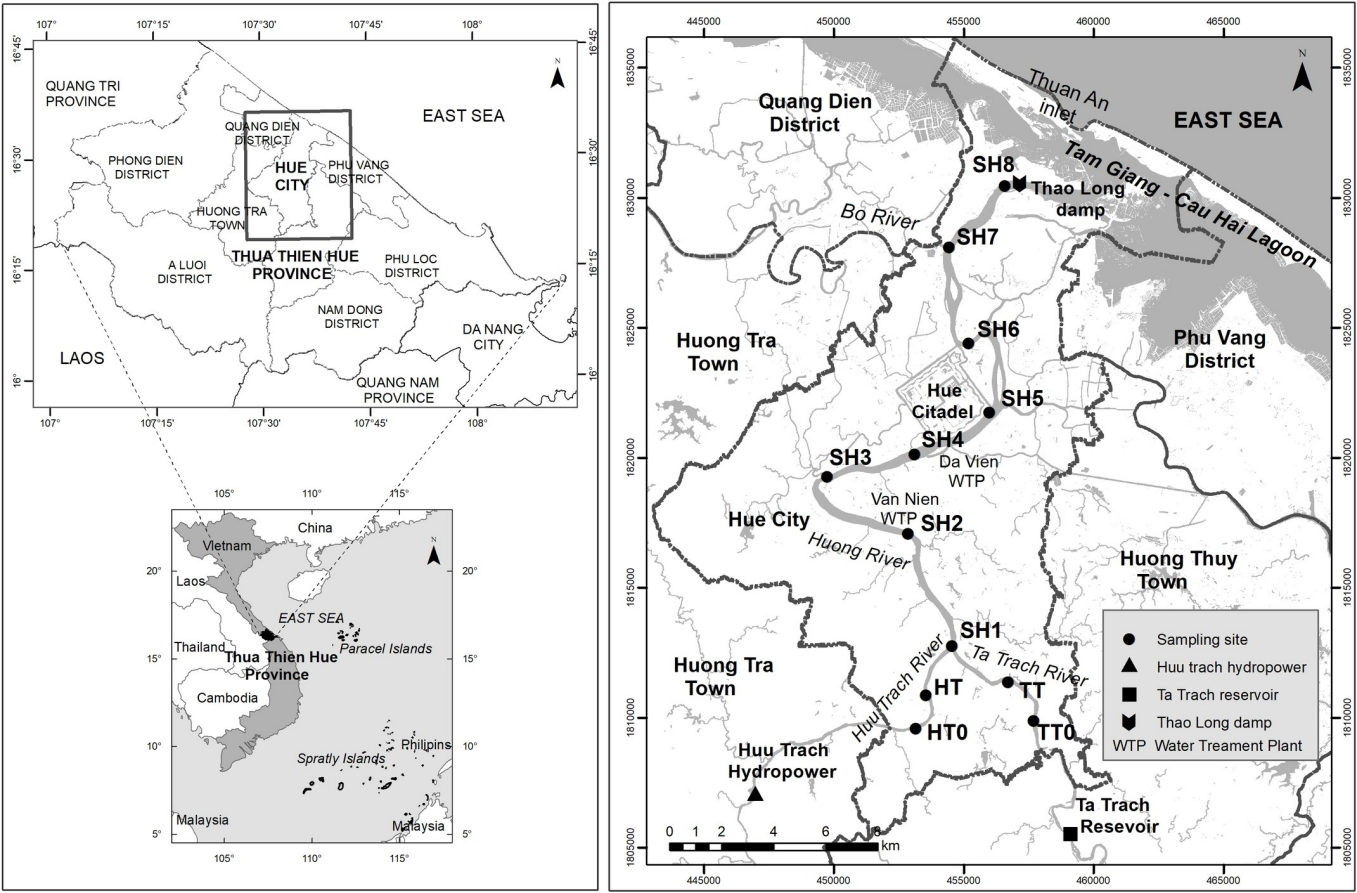
Study area and sampling sites in Huong river. Reprinted from thienhue.gov.vn/geditor.aspx?mapid=10528 under a CC BY license, with permission from Center for monitoring and operating smart cities—Department of Information and Communications—Thua Thien Hue Province, original copyright 2021.

Hue City (belonging to Thua Thien Hue province) was the ancient capital of Vietnam under the governing of the Nguyen Dynasty lasted from 1802 to 1945 and had been the political and cultural center in Central Vietnam since then. It is the noted sight-seeing resort that was registered as a World Culture Heritage since 1993. Huong river with a catchment area of 2830 km^2^ and a population of 540,000 in its basin is formed from two branches (Ta Trach and Huu Trach) originating from the mountains in the west of the province and combining at Tuan confluence. The main part of the river with 32 km length divides the city into two parts on its flowing way: north part (old city) and south part (new city), and meets Bo river at Sinh confluence (far from Hue city 15 km West), finally goes to Tam Giang-Cau Hai lagoon (running along the seaside) and then to the East sea at Thuan An outlet (Fig 1). The average width and depth of the main river part are 200 m and 2–8 m, respectively. Binh Dien hydro-power plant with a capacity of 423.7 million m^3^, located upstream of Huu Trach branch, has been operated since 2009. Ta Trach reservoir, with a capacity of 646 million m^3^, located upstream of Ta Trach branch, has been built for flood control purpose since 2013. A damp (Thao Long damp) has been built at the mouth area of the river in 2006 to prevent saline intrusion from the sea via the lagoon. Huong river is the most important surface water source used for different activities such as domestic activities, industries, irrigation, navigation, tourism, aquaculture, etc. in the province. Van Nien and Gia Vien are now two water intakes for two water treatment plants in the city. Wastewaters discharged into the river, floods in the wet season (September–December), and saline intrusion in the dry season (January–August) are environmental concerns to the river basin. Air temperature in the province is in the range of 21–38°C and 24.8°C on average. The annual average rainfall in the province is from 2700 mm to 3800 mm annually with a predominance of 60% in wet season. The river average flow was from 428 m^3^/s (in the dry season) to 553 m^3^/s (in the wet season), responding to the median flow from 189 m^3^/s to 214 m^3^/s, respectively (calculated from monitoring data in the years 2014–2016).

### Collection of water quality data

The water quality dataset used in this study is a seven-year monitoring data (2014–2020). It was divided into two sets: the dataset of the year 2014–2016 was used for WQI procedure development, while the dataset of 2017–2020 was employed for testing the WQI procedure developed and assessing the water quality of the Huong river. The water quality monitoring program was performed by the Institute of Natural Resources, Environment, and Biotechnology (IREB), Hue University, under the support of the Ministry of Training and Education, Vietnam. The water quality data were in the form of monthly data in reference to surface water samples collected every month at 11 monitoring sites (Hto, HT, Tto, TT, SH1 –SH3, and SH5 –SH8 shown in Fig 1 over a period of 3 years (2014–2016). Fourteen parameters that were routinely monitored were: temperature, pH, electrical conductivity (EC), total suspended solids (TSS), dissolved oxygen (DO), 5-day-biochemical oxygen demand (BOD), chemical oxygen demand (COD), ammonium (N-NH_4_), nitrate (N-NO_3_), phosphate (P-PO_4_), total coliform (TC), total dissolved iron (Fe), the river velocity and flow rate. Several total dissolved heavy metals (Hg^II^, Cd^II^, As^III,V^, Cr^VI^, Pb^II^, Cu^II^, Zn^II^) and organochlorine pesticides (DDTs, HCHs) were monitored one or two times per year.

The river water quality has also been quarterly monitored (in February, May, August and November) at ten sampling sites (HT, TT, and SH1 –SH8, Fig 1) by the Center for Natural Resources and Environment Monitoring (CREM) under the support of Thua Thien Hue Province–People Committee in the year of 2017–2020. The monitored parameters were the same as mentioned above.

Analytical methods for water quality parameters were adopted from Standard Methods for the Examination of Water and Waste Water [41]. Quality assurance and quality control procedures were conducted during the monitoring or analysis to confirm the data quality. Quality control consists of revising repeatability, trueness, linearity, limit of detection (LOD) and blank were routinely undertaken to confirm confidence of the monitoring/analysis results [41].

### Procedure of WQI development

**Scheme 1:**
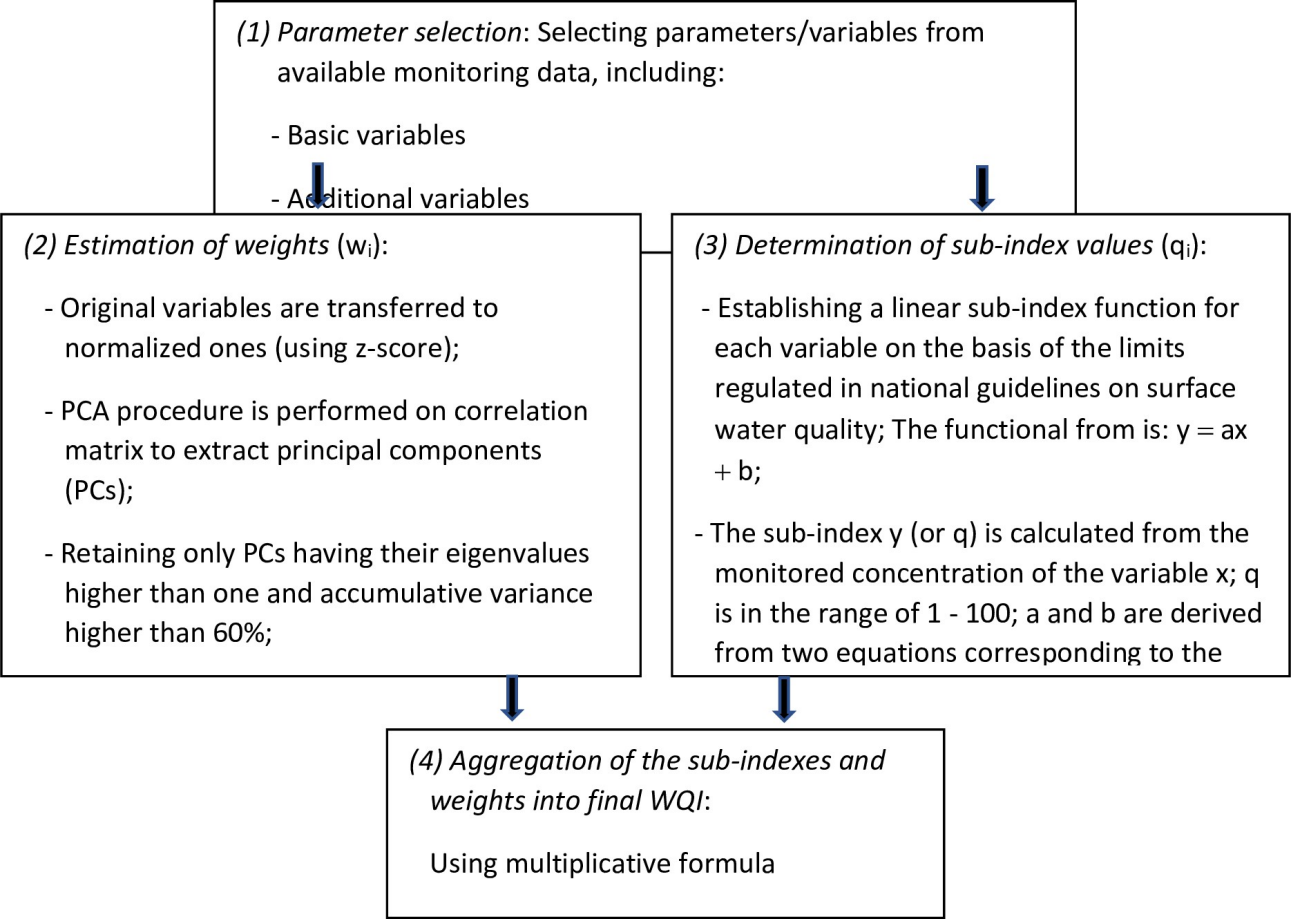
Procedure for the river WQI development.

The procedure of WQI development conducted in this study is described in Scheme 1.

- *Parameter selection*: Ten basic parameters (pH, EC, TSS, DO, BOD, COD, N-NH4, N-NO3, P-PO4, TC) and one additional parameter (Fe) were selected for the river WQI development. The parameters pH, EC, TSS and DO presents physical characteristics of the river. The parameters BOD, COD and N-NH4, N-NO_3_, P-PO_4_ indicates organic pollution and eutrophication levels of the river, respectively. The parameter TC describes fecal bacteria pollution level of the river. Iron is commonly occurred in the river waters due to erosion and washing from the soil in river basins and therefore, it is selected as an additional parameter in the WQI model. The heavy metals and organochlorides were not selected for the river WQI development, because their concentrations (collected from the available monitoring data) were very low, i.e. lower than the detection limit (LOD) or much lower than the limits of national guidelines on surface water quality [42] set up by Vietnam Ministry of Natural Resources and Environment/MONRE. The data set of the 11 parameters collected from IREB in the years 2014–2016 was used for the river WQI development. The original data set of 11 water quality parameters is supplied in S1 Data.

The data set of the 11 parameters (n = 11) collected from CREM in the year 2017–2020 (S2 Data) was used for testing the proposed WQI model and assessing the river water quality.

- *Estimation of weights*:

Principle component analysis method can ideally reduce the dimensionality of a multivariate data set while still maintaining its original structure to the maximum extent possible and thus it is often used while dealing with environmental data. The PCA reduces the total number of original variables to a smaller data set of new variables (factors or components) while preserving the variability with a minimal loss of information. The PCA method helps to extract the components/factors from the correlation matrix, necessary to explain the variance structure through linear combinations of the original variables [35]. For the PCA calculation, original variables are commonly transferred to normalized variables, which have zero mean and unit variance, to remove the effects of the variable unit and scale [35]. The eigenvalue of each component (or factor) is the amount of variance in the data set which is accounted for (or explained) by the component. The PCA calculation also gives the factor loading for each variable. Each factor loading represents the degree of contribution of the variable to the formation of the factor. The variables with the highest factorial load are considered of greater importance and should influence more on the factor [11,35]. In this study, the communality, which is a sum of square loadings of retained principal components (PCs) for each variable, was used for the calculation of the weight in the WQI procedure. The variable with the highest communality is considered of the most importance and vice versa. The PCA calculations were performed by using the free software R, version 4.0.3/64-bit (10-10-2020), module R-Studio and package Factoextra (version 1.0.7).

- *Determination of sub-index values*:

For convenience to WQI users in defining the sub-index of each selected parameter (or variable), linear sub-index functions are established based on the permissible limits from the National Technical Regulations on Surface Water Quality (QCVN 08:2015-MT/BTNMT) [42] set up by Vietnam Ministry of Natural Resource and Environment (MONRE) in 2015. The linear functional form for each variable (x) is:

y=a×x+b (Eq 2)

where y (or q) is sub-index calculated from the monitored concentration of the variable x;

a and b are derived from the two linear equations:

100=a+b×(limitofclassA1) (Eq 3)

where y = 100 corresponding to the best quality for variable x (≤ the limit of class A1 indicated in the regulation);

1=a+b×(limitofclassB2) (Eq 4)

where y = 1 corresponding to the worst quality for variable x (≥ the limit of class B2 in the regulation).

The water quality limits regulated for the selected parameters extracted from QCVN 08:2015-MT/BTNMT are shown in **Table 1**.

**Table 1:** Descriptive statistics for the 11 selected parameters (n = 396) ^(*)^.

	pH	EC	TSS	DO	BOD	COD	N-NH_4_	N-NO_3_	P-PO_4_	Fe	TC
	-	μS/cm	mg/L	mg/L	mg/L	mg/L	mg/L	mg/L	mg/L	mg/L	MPN/ 100 mL
**min**	5.4	16	1	4.4	2	5	0.07	0.1	0.05	0.19	130
**max**	7.2	955	76	7.8	9	22.6	0.42	0.7	0.17	0.88	7500
**mean**	6.4	70	13	6.2	3.6	10	0.16	0.32	0.08	0.43	1618
**STDV**	0.3	93	12	0.5	1.3	4	0.06	0.11	0.03	0.11	1177
**median**	6.3	49	8	6.2	3	9.0	0.15	0.3	0.08	0.42	1500
**MAD**	0.2	14	4	0.3	1	3.1	0.03	0.1	0.02	0.08	1020
**quartile 1**	6.2	38	5	5.8	3	6.5	0.12	0.22	0.06	0.35	480
**quartile 3**	6.5	70	14	6.5	5	13.0	0.2	0.4	0.1	0.51	2525
**CV (%)**	5	133	99	8	35	40	35	34	33	26	73
**CL95%**	0.03	9	1.2	0.05	0.12	0.4	0.01	0.01	0.01	0.01	116
**QCVN 08: A1**	6–8.5	1153	20	≥ 6	4	10	0.3	2	0.1	0.5	2500
**A2**	6–8.5	1538	30	≥ 5	6	15	0.3	5	0.2	1	5000
**B1**	5.5–9	3077	50	≥ 4	15	30	0.9	10	0.3	1.5	7500
**B2**	5.5–9		100	≥ 2	25	50	0.9	15	0.5	2	10000

^(*)^ QCVN 08: National technical regulation on surface water quality (QCVN 08-MT:2015/BTNMT); Class A1: Use for domestic water supply (after ordinary treatment), aquatic animals conservation and other uses as Class A2, B1, B2; Class A2: Use for domestic water supply (after suitable treatment) and other uses as Class B1, B2; Class B1: Use for irrigation or other purposes requiring the same water quality, and other uses as Class B2; Class B2: Use for water transportation and other uses requiring low water quality.

STDV—standard deviation; MAD—median absolute deviation; quartile 1—25th percentile; quartile 3—75th percentile; CV—coefficient of variation; CL95% - 95% confidence limit.

The DO concentrations higher than saturation indicate over algal synthesis in eutrophic waters, leading to a reduction in water quality. Saturated DO concentration at 20°C and the air pressure of 760 mmHg is 9 mg/L. This means that the sub-index (y) equals to 100 for the DO concentrations in the range of 6–9 mg/L (i.e. from the limit A1 to saturation with accepting that the lowest river water temperature was 20°C). In case the DO concentration is lower than 6 mg/L, the sub-index linear function for the parameter DO is determined following Eqs 3 and 4. If the DO concentration is over 9 mg/L (over saturation), a and b are derived from two equations:

100=a+b×9 (Eq 5)

and1=a+b×12. (Eq 6)

The pH limits in class A1 and A2 stated in the regulation range from 6 to 8.5, responding to the sub-index of 100. In the case of pH lower than 5.5 (limit B1) or higher than 9 (limit B2), the sub-index is equal to 1. This means that there are two sub-index functions for the parameter pH. Due to the parameter EC is not regulated in the QCVN 08:2015-MT/BTNMT [42], the sub-index linear function for the EC is established based on the limits for the parameter TDS required in the other regulations with approximately accepting that [43].


$$\text{TDS}\;\left(\text{mg}/\text{L}\right)=0.65\times \text{EC}(\mu \text{S}/\text{cm})$$ (Eq 7)

According to National Technical Regulations on Drinking Water Quality (QCVN 01:2009/BYT) [44] set up by the Vietnam Ministry of Health, the limit for TDS is lower than 1000 mg/L, approximate to EC < 1538 μS/cm that responds to the sub-index (y) of 100; According to National Technical Regulations on Water Quality for Irrigation (QCVN 39:2011/BTNMT) [45] set up by Vietnam MONRE, the limit for TDS is lower than 2.000 mg/L, approximate to EC < 3077 μS/cm that responds to the sub-index of (y) of 1. This means that in the sub-index linear equation for the EC, a and b are derived from two equations:

100=a+b×1538 (Eq 8)

and1=a+b×3077 (Eq 9)

- *Aggregation of the sub-index values into final WQI*:

Multiplicative method using formula Eq 1 mentioned above to calculate final WQI. Where, q_i_ is the parameter sub-index, ranging from 1 (the worst quality) to 100 (the best quality); w_i_ is the parameter weight defined from the PCA procedure, ranging from 0 to 1; sum of the weights equals to one.

### Water quality assessment basing on WQI grade

The grades representing the river water quality vary from 1 to 100. The classification of the river water quality, based on the WQI values, in this study is similar to the classification regulated in the VN-WQI model [30] (see S1 Text), as follows: grades 91–100 (EXCELLENT, color BLUE); 76–90 (GOOD, color GREEN); 51–75 (MODERATE, color YELLOW); 26–50 (POOR, color ORANGE); 10–25 (VERY POOR, color RED); < 10 (HIGHLY POLLUTED, color BROWN).

The proposed WQI was then applied to evaluate the river water quality employing the dataset in the years 2017–2020. The river water quality evaluations resulting from the proposed WQI were compared with the NSF-WQI and VN-WQI in several critical cases (the parameter concentrations above or below the limits) to examine ambiguity and eclipsing of the WQI indices in the river water quality reflection. The NSF-WQI is an index calculated according to either multiplicative formula (Eq 1) or additive one (Eq 2) with nine selected parameters (n = 9) consisting of temperature change (ΔT), pH, Tur (turbidity), TS (total solids), DO, BOD_5_, N-NO3, P-PO4 and fecal coliform (NSF-WQI, 1970). It includes the parameter weights:

$$\text{WQI}=\sum_{i=1}^{9}{\text{w}}_{\text{i}}{\text{q}}_{\text{i}}$$ (Eq 10)

In this study, the NSF-WQI was calculated according to both the formulas (Eqs 1 and 10).

The original data set of the nine water quality parameters mentioned above and the results obtained from the NSF-WQI calculation are supplied in S3 Data. The parameter subindex (q_i_) was derived from the respective rating curve. DO concentration (mg/L) at a given water temperature (extracted from S2 Data) was converted into DO saturation (%) to define the subindex for parameter DO. The parameter ΔT was obtained by subtracting the upstream temperature from the temperature downstream and recording the result as temperature change (°C). The parameter TS was accepted to be the sum of TDS and TSS: TS = TDS + TSS, where TDS (total dissolved solids) concentration was estimated by: TDS (mg/L) = 0.65 × EC (μS/cm); the parameters EC and TSS were extracted from S2 Data. Fecal coliform concentration was replaced by the total coliform (TC) concentration for the NSF-WQI calculation. The relative weights for the parameters (w_i_ in parenthesis) are as follows (in decrease order of the w_i_): DO (0.17), TC (0.16), pH (0.11), BOD (0.11), ΔT (0.10), N-NO3 (0.10), P-PO4 (0.10), Tur (0.08), TS (0.07).

The VN-WQI is an index without the parameter weight, meaning that the selected parameters have equal weight (weights are all equal to one). The sub-index value for each parameter is defined from the normalized scales given in the appropriate table. The sub-index for the parameter DO is derived from a given equation with monitored water temperature. The final VN-WQI value is calculated with both multiplicative and additive methods (the VN-WQI model is supported in S1 Text). In this study, the index VN-WQI applied to the river was calculated from eight parameters (n = 8): pH (belongs to Group I); DO, BOD, COD, N-NH4, N-NO3 and P-PO4 (Group IV) and TC (Group V). The heavy metals including As, Cd, Pb, Cr^VI^, Cu, Zn, Hg (Group III) and organochlorides such as aldrin, BHCs, dieldrin, DDTs, heptachlor and heptachlor epoxide (Group II) were not selected for the VN-WQI calculation because their concentrations monitored in the river samples in the years 2017–2020 were lower than the detection limits (LODs) or much lower than the limits regulated by Vietnam MONRE (QCVN 08-MT:2015/BTNMT) [42].

## Results and discussion

### Application of principal component analysis to define weights

Arief et al. [4] recommended a minimum of 150–300 cases to be studied for principal component analysis (PCA) and factor analysis (FA) to achieve reliable results. This study satisfies this criterion as it uses monthly data of the 11 parameters at 11 monitoring sites in three years (2014–2016) i. e. 396 cases (= 11 × 12 × 3).

Descriptive statistics, processed from Microsoft-Excel using Real Statistics tool, are described in Table 1. The National Technical Regulation on Surface Water Quality set up by Vietnam MONRE in 2015 (QCVN 08-MT:2015/BTNMT) [42] is also included in Table 1 to indicate the permissible limits of the parameters that are used for establishing the linear sub-index functions. These results are also used for a preliminary overview of the river water quality which will be discussed in the next sections.

The PCA procedure was performed on the Pearson correlation matrix of the 11 selected variables, extracting 11 new components with their own eigenvalues. The criterion to decide the number of components to be retained is adopted from the previous WQI developers [11,24,46]. Ideally, the retained components should have the following characteristics: (i) Cumulative contribution to the overall variance is greater than 60%; (ii) Associated eigenvalues are higher than one. The component eigenvalue higher than one should be retained as it explains at least more one original variable in the data set; If below 1, the new component does not provide more information than the original variable and, therefore, is of little interest [24,35]. **Table 2** presents the eigenvalues from the PCA, the percentage of variance explained by each component and the cumulative variance. The cumulative variance for the first three (3) principal components (Comp.1 –Comp.3), which is equal to 67.0%, satisfies the recommendations and was adopted to use for the calculation of the parameter weights in the proposed WQI in the present work. The 33% of the remaining total variance of the data was assigned to ‘noise’ or background variation.

**Table 2:** Eigenvalues, variance percentages and cumulative variances for the components.

Component (Comp.)	Eigenvalue	Variance percent	Cumulative variance
**Comp.1**	4.94	44.89	44.89
**Comp.2**	1.40	12.76	57.64
**Comp.3**	1.03	9.39	67.04
**Comp.4**	0.87	7.91	74.94
**Comp.5**	0.64	5.81	80.76
**Comp.6**	0.45	4.12	84.88
**Comp.7**	0.44	4.02	88.90
**Comp.8**	0.39	3.58	92.48
**Comp.9**	0.35	3.18	95.66
**Comp.10**	0.31	2.78	98.44
**Comp.11**	0.17	1.56	100.00

**Fig 2:**
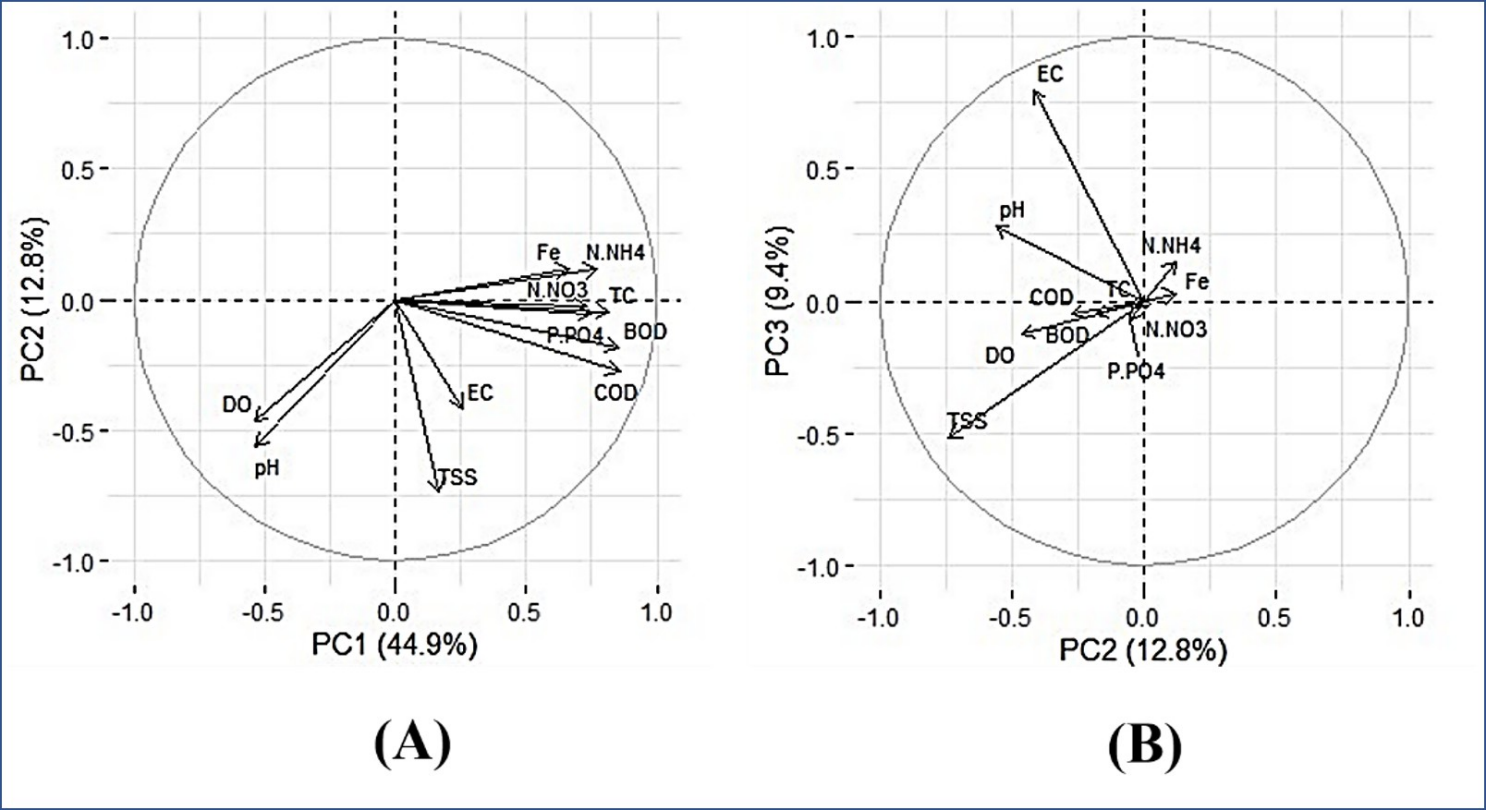
Loading plots: (A) PC1 × PC2 and (B) PC2 × PC3.

The PCA outputs helped evaluate the variable level of explanation relevant to the analysis, meaning which variables are responsible for the patterns seen among the observations. The factorial load from the PCA is the correlation of the variable with the respective component. A positive value of the factorial load demonstrates a positive correlation with the component of the variable. If it is negative, this correlation is negative. In other words, the variable has a direction of variation opposite to that of the construct. **Table 3** shows factor loadings of the variables on the first three principal components (PC1 –PC3). The loading plots for PC1 × PC2 and PC2 × PC3 are shown in Fig 2.

**Table 3:** Factor loadings for the variables on the first three principal components.

No.	Parameters	PC1	PC2	PC3
**1**	pH	-0.536	-0.564	0.281
**2**	EC	0.257	-0.418	0.796
**3**	TSS	0.168	-0.740	-0.519
**4**	DO	-0.542	-0.466	-0.124
**5**	BOD	0.853	-0.186	-0.052
**6**	COD	0.857	-0.277	-0.047
**7**	N-NH_4_	0.773	0.118	0.151
**8**	N-NO_3_	0.733	-0.024	-0.028
**9**	P-PO_4_	0.750	-0.056	-0.072
**10**	Fe	0.661	0.120	0.030
**11**	TC	0.816	-0.046	-0.014

The results in Table 3 and loading plots in Fig 2 indicated that:

- Principal component 1 (PC1) explains 44.9% of the total variability of the data and is the most important in the analysis. Liu et al. [47] classified the significant loadings as ‘‘strong” (absolute loading value > 0.75), ‘‘moderate” (0.50 to 0.75), and ‘‘weak” (0.30 to 0.50). This classification was adopted by Ouyang [48] and Singh et al. [49]. Thus, the PC1 accounts for the nine variables related to water quality that emerged with strong to moderate loadings (higher than ± 0.5). The TSS and EC variables had very weak loadings on PC1, accounting for 0.168 and 0.257, respectively. Most of these nine variables have positive correlations with the PC1, except for variables pH and DO having negative correlations (opposite variation directions again the positive direction of the PC1).
- PC2 explains 12.8% of the total variance of the data and mainly accounts for two (2) variables with negative correlation: TSS (-0.740) and pH (-0.564).
- PC3 explains only 9.4% of the total variance of the data and mainly accounts for two (2) variables: EC (0.796) and TSS (-0.519).

The next step for the WQI formulation is to define the degree of relevance of each variable (or parameter) that helps establish the relative weight (w_i_). From factor loading values in Table 3, the squared loadings and then the communality values, which represent the amount of variance explained by each variable in the factorial solution, are calculated. Table 4 presents the squared loadings and communality values for the variables on three principal components (PC1 –PC3). The largest communality value in the column is for the parameter EC (0.875), providing the greatest relative weight (w_i_) and the smallest communality value for Fe (0.452), giving the smallest relative weight. Then, the procedure to define the relative weight (w_i_) of each parameter is easily conducted by dividing its communality value by the sum of the communality values in the column (7.374). Using the communality values and the procedure defined in this study, the relative weight (w_i_) for each parameter is calculated and exhibited in Table 4. The sum of the eleven weights adds to one (1.00).

**Table 4:** Square loadings, communality values and relative weights for the parameters.

Parameter	PC1	PC2	PC3	Communality	Relative weight (w)
**pH**	0.287	0.318	0.079	0.684	0.10
**EC**	0.066	0.175	0.634	0.875	0.12
**TSS**	0.028	0.548	0.270	0.846	0.11
**DO**	0.294	0.217	0.015	0.526	0.07
**BOD**	0.728	0.035	0.003	0.766	0.10
**COD**	0.734	0.077	0.002	0.813	0.11
**N-NH**_**4**_	0.597	0.014	0.023	0.633	0.09
**N-NO**_**3**_	0.537	0.001	0.001	0.538	0.07
**P-PO**_**4**_	0.563	0.003	0.005	0.571	0.08
**Fe**	0.437	0.014	0.001	0.452	0.06
**TC**	0.667	0.002	0.000	0.669	0.09
**Eigenvalue** ^(*)^	**4.937**	**1.403**	**1.033**		

^(*)^ Eigenvalue equals to the sum of the squared loadings in the respective column. The sum of the eigenvalues equals to the sum of the communality values for the eleven parameters.

Thus, the PCA helped to define the weight of importance for each parameter, independent of subjective assessments. The next step is to transform the concentration monitored for each parameter, into dimensionless grade (sub-index q_i_), to calculate the WQI value for each water sample.

### Linear functions to transform dimensional water quality parameters into dimensionless sub-indices

**Fig 3:**
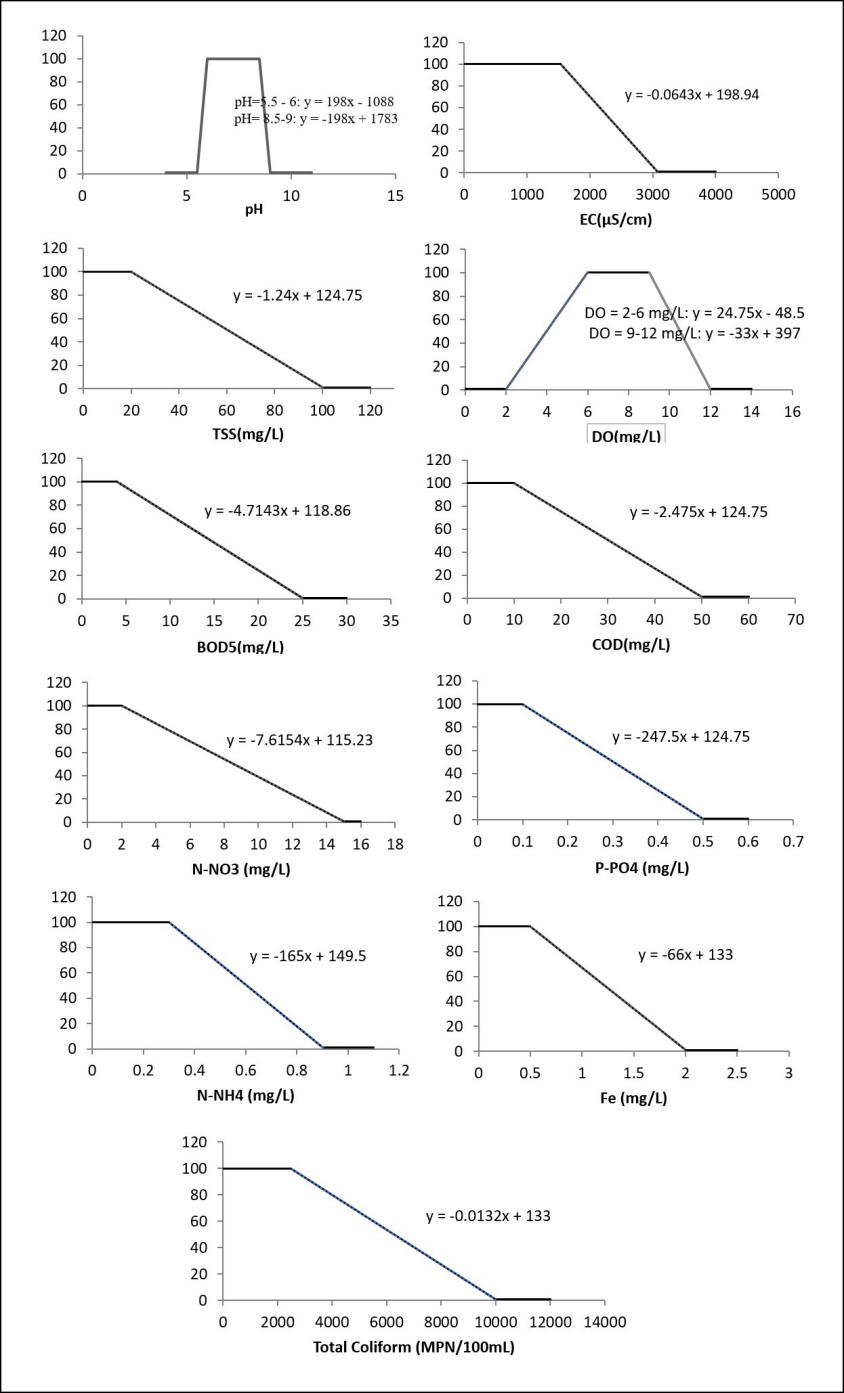
The linear curve and equation(s) for each parameter to transform the concentration x (in the abscissa) into the grade y (or sub-index q) in the ordinate.

Linear curves with the monitored concentrations of the parameters in the abscissa and the grades (sub-indices q) ranging from 1 to 100 in the ordinate were developed using the limits for surface water quality regulated by Vietnam MONRE (QCVN 08-MT:2015/BTNMT [42], shown in Table 1) and the procedure described above. Fig 3 shows the curves (concentration versus grade) and linear equations for the eleven parameters: pH, EC, TSS, DO, BOD, COD, N-NH_4_, N-NO_3_, P-PO_4_, Fe and TC.

### Application of the WQI to Huong river in Thua Thien Hue province

In the period 2014–2020, there has been no publication on WQI development to assess the quality of Huong river. The proposed WQI index was, for the first time, applied to evaluate Huong river water quality in the period of 2017–2020. The final WQI values were calculated using the multiplicative formula with the respective weights and sub-indices (Eq (2)). The results of the WQIs were shown in Table 5.


$$WQI=\prod_{\text{i=1}}^{\text{n}}{\text{q}}_{\text{i}}^{{\text{w}}_{\text{i}}}=q_{pH}^{0.10}\times q_{pH}^{0.12}\times q_{pH}^{0.11}\times q_{pH}^{0.07}\times q_{pH}^{0.10}\times q_{pH}^{0.11}=q_{pH}^{0.10}\times q_{EC}^{0.12}\times q_{TSS}^{0.11}\times q_{DO}^{0.07}\times q_{BOD}^{0.10}\times q_{COD}^{0.11}\times q_{N.NH4}^{0.09}\times q_{N.NO3}^{0.07}\times q_{P.PO4}^{0.08}\times q_{Fe}^{0.06}\times q_{TC}^{0.09}$$ (Eq 11)

**Table 5:** The river WQI values for the ten sampling stations in the years 2017–2020.

Month (year)^(*)^	TT	HT	SH1	SH2	SH3	SH4	SH5	SH6	SH7	SH8
**Feb. (2017)**	98	100	100	100	99	100	100	98	98	100
**May (2017)**	97	97	95	98	99	100	100	97	98	98
**Aug. (2017)**	98	97	99	99	99	99	98	98	99	97
**Nov. (2017)**	100	100	100	100	99	100	100	100	100	100
**Feb. (2018)**	97	99	99	99	99	100	99	98	98	98
**May (2018)**	96	98	97	99	98	99	98	97	94	100
**Aug.1 (2018)**	91	96	91	97	97	96	98	95	96	96
**Aug.2 (2018)**	97	97	98	99	99	98	99	97	96	97
**Nov. (2018)**	100	97	99	100	100	100	97	95	97	100
**Feb. (2019)**	99	100	99	99	100	100	100	100	99	100
**May1 (2019)**	94	100	98	100	100	100	100	98	100	99
**May2 (2019)**	98	96	97	97	98	99	98	98	99	96
**Aug. (2019)**	100	99	99	97	99	99	66	98	96	100
**Nov. (2019)**	98	98	98	99	99	100	99	100	98	98
**Feb. (2020)**	98	97	98		97		98	98	97	98
**May1 (2020)**	96	99	100		100		100	100	100	96
**May2 (2020)**	97	98	98		97		99	99	95	92
**Aug. (2020)**	97	97	95		99		99	98	98	98
**Nov. (2020)**	72	65	81		80		82	66	80	84

^(*)^ Aug.1, Aug.2 and May1, May2 are monitoring sessions 1 and 2 in August and May, respectively.

The calculations presented in the spreadsheet (the river water quality data set in the years 2017–2020 with total data of 1980 (180 cases × 11 variables) (S2 Data) indicated 96.6% of the set had concentrations below the A1 limit (89.1%) and A2 limit (7.5%); 3.2% of the set had concentrations above the B1 limit (2.4%) and B2 limit (0.8%); and 0.2% of the set had concentrations above B2 limit. Based on these results, it is expected that around 97% of WQI values were of grades EXCELLENT or GOOD and around 3% of grades MODERATE or POOR. These results are quite the same from the river WQI values: 97.8% of WQI values were of grades EXCELLENT or GOOD and 2.2% of grade MODERATE.

Generally, the river water quality was rather good in terms of the WQI: 97.8% of grades EXCELLENT or GOOD. Discharging water from the Ta Trach reservoir into the river in the flooding season due to heavy rainfall (in November 2020) led to an increase in the TSS and Fe concentrations and a decrease in the DO concentrations. Consequentially, the WQI values in these cases were decreased (the DO, TSS, and Fe concentrations, and the WQI values for the monitoring session in Nov. 2020 are shown in Table 6). Besides, rather high concentrations of the total coliform (TC) for the site SH5 in Aug. 2019 (15000 MPN/100 mL, above the limit B2) and site SH6 in Nov. 2020 (4600 MPN/100 mL, above the limit A2) also contributed to the decrease in the WQI values (= 66, appropriate to the grade MODERATE). These results indicated that the proposed WQI index was a sensitive reflection of the river water quality. For comparison, the index NSF-WQI and VN-WQI were also calculated for the monitoring session in Nov. 2020 (also shown in Table 6).

**Table 6:** The DO, TSS, Fe and TC concentrations, WQI, NSF-WQI and VN-WQI values, and the respective grades for the monitoring session in Nov. 2020 ^(*)^.

	DO	TSS	Fe	TC	WQI value-grade	NSF-WQI_M_ value-grade	NSF-WQI_A_ value-grade	VN-WQI value-grade
Site	(mg/L)	(mg/L)	(mg/L)	(MPN/ 100 mL				
**TT**	5	56	1.99	43	72 -M	68 -M	72 -G	100 -E
**HT**	5.2	76.5	2.23	240	65 -M	57 -M	69 -G	100 -E
**SH1**	5.1	51.5	1.85	240	81 -G	65 -M	72 -G	100 -E
**SH3**	5.3	66.5	1.81	93	80 -G	61 -M	73 -G	100 -E
**SH5**	5.4	66	1.71	93	82 -G	68 -M	74 -G	100 -E
**SH6**	5.2	65	2.07	4600	66 -M	50 -P	67 -M	89 -G
**SH7**	5.6	59.5	1.86	1100	80 -G	60 -M	69 -M	100 -E
**SH8**	5.5	61	1.64	1500	84 -G	61 -M	70 -M	100 -E

^(*)^ The DO, TSS, Fe concentrations were extracted from the S1 Data.

The WQI values were extracted from Table 5.

The limits from the Vietnam MONRE regulation on surface water quality (QCVN 08-MT:2015/BTNMT, extracted from Table 1): DO = 6 mg/L (limit A1) and 5 mg/L (limit A2); TSS = 50 mg/L (B1) and 100 mg/L (B2); Fe = 1.5 mg/L (B1) and 2 mg/L (B2); total coliform (TC) = 2500 MPN/100 mL (limit A1) and 5000 MPN/100 mL (A2).

NSF-WQI_M_: NSF-WQI of multiplicative formula.

NSF-WQI_A_: NSF-WQI of additive formula.

NSF-WQI values are classified into following water quality grades: 91–100 (EXCELLENT); 71–90 (GOOD); 51–70 (MODERATE); 26–50 (POOR); 0–25 (VERY POOR).

Abbreviations of grades: E: Excellent; G: Good; M: Moderate; P: Poor.

The results from **Table 6** show that compared with the proposed WQI, the NSF-WQI_M_ and NSF-WQI_A_ values are remarkably lower. The reason for that is the relative weights for parameters DO and TC in the NSF-WQI are higher than that in the index WQI. Although there are four of eight cases that the water quality grades from the NSF-WQI_A_ and proposed WQI are the same, the values of the two indexes are significantly different (p = 0.044; paired-t-test). In addition, the differences in the river water quality reflection between the NSF-WQI and the proposed WQI occurred due to differences in the selected parameters and number of the parameters incorporated in the indexes. Collating the results of these indexes (NSF-WQI_M_, NSF-WQI_A_ and the proposed WQI) with the values monitored for the parameters in comparison with the limits from Vietnam MONRE regulations, the proposed WQI index is more suitable in the river water quality assessment. Also, compared with the VN-WQI, the proposed WQI has no ambiguity and eclipsing due to representing the actual state of overall water quality. The reason for the less representative of the VN-WQI is that the parameters TSS and Fe are not integrated into the VN-WQI calculation. Another issue of the VN-WQI is that it does not reflect the impact of saline intrusion on the water quality because the parameter related to dissolved solids such as EC or TDS is not integrated into the index.

## Conclusion

A comprehensive and simple procedure to develop the WQI using the available monitoring data of Huong river water quality was proposed. Multivariable technique (PCA) was applied to objectively define relative weight (w_i_) for each water quality parameter, based on the set of communality values for the 11 selected parameters. The use of the limits from the national guideline on surface water quality for establishing the linear functions to transform the dimensional concentration into dimensionless sub-index (q_i_) for each parameter provided convenience for the WQI users. The multiplicative formula which operates the sub-index (q_i_) raised to a power (w_i_), or the weight of importance of each variable, allowed to calculate the final WQI values. Comparison between the river water quality evaluations resulting from the proposed index (WQI), with the index NSF-WQI and index issued by Vietnam Environment Agency (VN-WQI) in 2019 indicated the different classifications using the three indexes. The representative reflection of the actual state of the river general water quality in term of the WQI shows that the WQI avoided ambiguity and eclipsing occurred to the VN-WQI. Finally, the developed procedure and WQI could be used for the river quality assessment in the coming years as well as for practical applications on a local or regional scale.

## Supporting information

S1 Data Huong river water quality parameters monitored in the years 2017–2020. (XLSX)

S2 Data Huong river water quality parameters monitored in the years 2017–2020. (XLSX)

S3 Data Huong river water quality parameters monitored in November 2020 (used for NSF-WQI calculation). (XLSX)

S1 Text Decision No. 1460/QD-TCMT dated 12 November 2019, issued by Vietnam Environment Agency (VEA), regarding the promulgation of Technical Guidelines for calculation and publication of the Vietnam Water Quality Index (VN-WQI). (DOCX)

## References

[1] Brown RM, McClelland NI, Deininger RA and Tozer RG. A water quality index—do we dare? Water Sewage Works. 1970; 117:339–343.

[2] Abbasi T and Abbasi SA. Water Quality Indices. Elsevier. 2012.

[3] Mehmet ATK, Sevgili H. Parameters selection for water quality index in the assessment of the environmental impacts of land-based trout farms. Ecol. Indic. 2014; 36:672–681. 10.1016/j.ecolind.2013.09.034.

[4] Arief DS, Nitin M, Abdullah GY and Perera BJC. Development of river water quality indices—a review. Environ. Monit. Assess. 2016; 188:58. doi: 10.1007/s10661-015-5050-0 26707404

[5] Siddhant D and Ajay SK. Science mapping approach to critical reviewing of published literature on water quality indexing. Ecol. Indic. 2021; 128 (2021) 107862. doi: 10.1016/j.ecolind.2021.107862

[6] Bordalo AA, Teixeira R, Wiebe WJ. A water quality index applied to an international shared river basin: the case of the Douro River. Environ. Manage. 2006; 38:910–920. doi: 10.1007/s00267-004-0037-6 17039391

[7] Sanchez E, Colmenarejo MF, Vicente J, Rubio A, García MG, Travieso L, et al. Use of the water quality index and dissolved oxygen deficit as simple indicators of watersheds pollution. Ecol. Indic. 2007; 7:315–328. 10.1016/j.ecolind.2006.02.005.

[8] Tian Y, Jiang Y, Liu Q, Dong M, Xu D, Liu Y, et al. Using a water quality index to assess the water quality of the upper and middle streams of the Luanhe River, northern China. Sci. Total Environ. 2019; 667:142–151. doi: 10.1016/j.scitotenv.2019.02.356 30826675

[9] Ashok L, Sharma TC, Bibeault JF. A Review of genesis and evolution of Water Quality Index (WQI) and some future directions. Water. Qual. Expo. Health. 2011; 3:11–24. 10.1007/s12403-011-0040-0.

[10] Noori R, Berndtsson R, Hosseinzadeh M, Adamowski JF, Abyaneh MR. A critical review on the application of the National Sanitation Foundation Water Quality Index. Environ. Pollut. 2018; 244:575–587. doi: 10.1016/j.envpol.2018.10.076 30384063

[11] José BF and Iara BO. Development of a groundwater quality index: GWQI, for the aquifers of the state of Bahia, Brazil using multivariable analyses. Sci. Rep. 2021; 11:16520. doi: 10.1038/s41598-021-95912-9 34389745 PMC8363630

[12] House MA. A water quality index for river management. Water Environ. J. 1989; 3:336–344. 10.1111/j.1747-6593.1989.tb01538.x.

[13] Dojlido J, Raniszewski J, Woyciechowska J. Water quality index applied to rivers in the Vistula River basin in Poland. Environ. Monit. Assess. 1994;33(1):33–42. doi: 10.1007/BF00546659 24201699

[14] Cude CG. Oregon water quality index: a tool for evaluating water quality management effectiveness. J. Am. Water Resour. Assoc. 2001; 37(1):125–137. doi: 10.1111/j.1752-1688.2001.tb05480.x

[15] DoE (Department of Environment) Malaysia. Malaysia environmental quality report 2001. Department of Environment, Ministry of Science, Technology and Environment.

[16] Liou SM, Lo SL, Wang SH. A generalized water quality index for Taiwan. Environ. Monit. Assess. 2004; 96(1–3):35–52. doi: 10.1023/b:emas.0000031715.83752.a1 15327148

[17] Said A, Stevens DK, Sehlke G. An innovative index for evaluating water quality in streams. Environ. Manage. 2004; 34(3):406–414. doi: 10.1007/s00267-004-0210-y 15520897

[18] Almeida C, González S, Mallea M, González P. A recreational water quality index using chemical, physical and microbiological parameters. Environ. Sci. Pollut. Res. 2012; 19(8):3400–3411. doi: 10.1007/s11356-012-0865-5 22528988

[19] Swamee P, Tyagi A. Improved method for aggregation of water quality subindices. J. Environ. Eng. 2007; 133(2):220–225. 10.1061/(ASCE)0733-9372(2007)133:2(220).

[20] CCME (Canadian Council of Ministers of the Environment). Canadian water quality guidelines for the protection of aquatic life. CCME-Water Quality Index 1.0, Technical Report. 2001; Canadian Council of Ministers of the Environment, Winnipeg, MB, Canada.

[21] Terrado M, Barceló D, Tauler R, Borrell E, Campos SD, Barceló D. Surface water quality indices for the analysis of data generated by automated sampling networks. Trends Anal. Chem. 2010; 29(1):40–52. 10.1016/j.trac.2009.10.001.

[22] Joung HM, Miller WW, Mahannah CN, and Guitjens JC. A generalized water quality index based on multivariate factor analysis. J. Environ. Qual. 1979; 8(1):95–100. 10.2134/jeq1979.00472425000800010021x.

[23] Hanh PTM, Sthiannopkao S, Ba DT, Kim KW. Development of water quality indexes to identify pollutants in Vietnam’s surface water. J. Environ. Eng. 2011; 137(4): 273–283. 10.1061/(ASCE)EE.1943-7870.0000314.

[24] Mansi T, Sunil KS. Allocation of weights using factor analysis for development of a novel water quality index. Ecotoxicol. Environ. Saf. 2019; 183:109510. doi: 10.1016/j.ecoenv.2019.109510 31401332

[25] Prati L, Pavanello R, Pesarin F. Assessment of surface water quality by a single index of pollution. Water Res. 1971; 5(9):741–751. 10.1016/0043-1354(71)90097-2.

[26] Bascarón M. Establishment of a methodology for the determination of water quality. Boletin Informativo del Medio Ambiente. 1979; 9: 30–51.

[27] Štambuk-Giljanović N. Comparison of Dalmatian water evaluation indices. Water Environ. Res. 2003; 75(5):388–405. doi: 10.2175/106143003x141196 14587950

[28] Harkins RD. An objective water quality index. J. Water Pollut. Control Fed. 1974; 46(3):588–591. 10.2307/25038160. 4846806

[29] Bhargava DS. Use of a water quality index for river classification and zoning of Ganga River. Environmental Pollution Series B: Chemical and Physical. 1983; 6(1):51–67. 10.1016/0143-148X(83)90029-0.

[30] VEA Vietnam Environment Administration. Decision No. 1460 / QD-TCMT, dated 12 November 2019, Regarding the promulgation of Technical Guidelines for calculation and publication of the Vietnam Water Quality Index (VN_WQI).

[31] Gazzaz NM, Yusoff MK, Aris AZ, Juahir H, Ramli MF. Artificial neural network modeling of the water quality index for Kinta River (Malaysia) using water quality variables as predictors. Mar. Pollut. Bull. 2012; 64(11):2409–2420. doi: 10.1016/j.marpolbul.2012.08.005 22925610

[32] Vega M, Pardo R, Barrado E, Debán L. Assessment of seasonal and polluting effects on the quality of river water by exploratory data analysis. Water Res. 1998; 32:3581–3592. 10.1016/S0043-1354(98)00138-9.

[33] Yun Q, Kati WM, Yongshan W, Yuncong L. Surface water quality evaluation using multivariate methods and a new water quality index in the Indian River Lagoon, Florida. Water Resour. Res. 2007; 43(8):W08405, 10.1029/2006WR005716.

[34] Christiane C, Roberto T, Túlio APR, Renata TGS, Daniela AP. Water quality index using multivariate factorial analysis. Revista Brasileira de Engenharia Agrícola e Ambiental. 2010; 14(5):517–522.

[35] Daniel JD. Univariate, Bivariate, and Multivariate Statistics Using R. John Wiley & Sons. 2020.

[36] Abdelmonem A, Susanne M, Robin AD, Virginia AC. Practical Multivariate Analysis. Chapman & Hall/CRC, 6th Edition. 2020.

[37] Bhargava DS. Water quality variations and control technology of Yamuna River. Environ. Pollut. A. 1985; 37(4):355–376. 10.1016/0143-1471(85)90124-2.

[38] Dinius SH. Design of an index of water quality. J. Am. Water Resour. Assoc. 1987; 23(5):823–843. 10.1111/j.1752-1688.1987.tb02959.x.

[39] Smith DG. A better water quality indexing system for rivers and streams. Water Res. 1990; 24(10):1237–1244. 10.1016/0043-1354(90)90047-A.

[40] Juwana I, Muttil N, Perera BJC. Indicator-based water sustainability assessment—a review. Sci. Total Environ. 2012; 438(0):357–371. doi: 10.1016/j.scitotenv.2012.08.093 23022721

[41] SMEWW—Rice EW, Baird RB, Eaton AD, Clesceri LS. Standard methods for the examination of water & wastewater. Volume 22, American Public Health Association, American Water Works Association, Water Environment Federation. 2012.

[42] QCVN 08-MT:2015/BTNMT. National Technical Regulation on Surface Water Quality. Vietnam Ministry of Natural Resources and Environment. 2015.

[43] Roger NR. Introduction to environmental analysis. John Wiley & Sons Ltd. 2002.

[44] QCVN 01:2009/BYT. National Technical Regulation on Drinking Water Quality. Vietnam Ministry of Health. 2009.

[45] QCVN 39:2011/BTNMT. National Technical Regulation on Water Quality for Irrigation. Vietnam Ministry of Natural Resources and Environment. 2011.

[46] Nicoletti G, Scarpetta S, Boyland O. Summary indicators of product market regulation with an extension to employment protection legislation. OECD Economics Department Working Papers No. 226. 1999.

[47] Liu CW, Lin KH, Kuo YM. Application of factor analysis in the assessment of groundwater quality in a blackfoot disease area in Taiwan. Sci. Total Environ. 2003; 313:77–89. doi: 10.1016/S0048-9697(02)00683-6 12922062

[48] Ouyang Y. Evaluation of river water quality monitoring stations by principal component analysis. Water Res. 2005; 39:2621–2635. doi: 10.1016/j.watres.2005.04.024 15993926

[49] Singh KP, Malik A, Singh VK, Mohan D, Sinha S. Chemometric analysis of groundwater quality data of alluvial aquifer of Gangetic plain, North India. Anal. Chim. Acta. 2005; 550:82–91.

