# Supplementary material for: A comprehensive procedure to develop water quality index: A case study to the Huong river in Thua Thien Hue province, Central Vietnam
Source: PLoS One. 2022 Sep 15;17(9):e0274673. doi: 10.1371/journal.pone.0274673 (PMC9477372; doi:10.1371/journal.pone.0274673)
Supplement: S1 Text — (DOCX) [file pone.0274673.s004.docx]

**Supporting information**

**S1 Text**

**Decision No. 1460/QD-TCMT dated 12 November 2019, issued by Vietnam Environment Agency (VEA), regarding the promulgation of Technical Guidelines for calculation and publication of the Vietnam Water Quality Index (VN-WQI).**

1. **Guidelines for calculating** the water quality index (VN-WQI)

**Data requirements:** using reliable and available water quality data obtained from monitoring program conducted in local or regional area; QA/QC process must be regularly implemented.

1. **Selected parameters**: The parameters used to calculate VN-WQI are divided into 5 groups, including:

**Group I**: pH

**Group II**: pesticides, including Aldrin, BHC, Dieldrin, DDTs (p,p’-DDT, p,p’-DDD, p,p’-DDE), Heptachlor & Heptachlorepoxid

**Group III**: dissolved heavy metals, including As^III,V^, Cd^II^, Pb^II^, Cr^VI^, Cu^II^, Zn^II^ and Hg^II^

**Group IV**: organic and nutrient pollutants, including DO (dissolved oxygen), BOD5 (five-day biochemical oxygen demand), COD (chemical oxygen demand), TOC (total organic carbons), N-NH4 (ammonium), N-NO3 (nitrate), N-NO2 (nitrite), P-PO4 (phosphate)

**Group V**: bacterials, including Total coliform and E. coli.

The least numbers of the parameter groups used for the VN-WQI calculation must be three of the five groups, in which, in group IV, there are at least three parameters used for calculation.

1. **Calculation of parameter water quality index (WQI_SI_) or subindex for each parameter**

- Calculation for As^III,V^, Cd^II^, Pb^II^, Cr^VI^, Cu^II^, Zn^II^, Hg^II^, BOD_5_, COD, TOC, N-NH4, N- NO2, N-NO3, P-PO4, Total coliform, and E. coli was as follows:


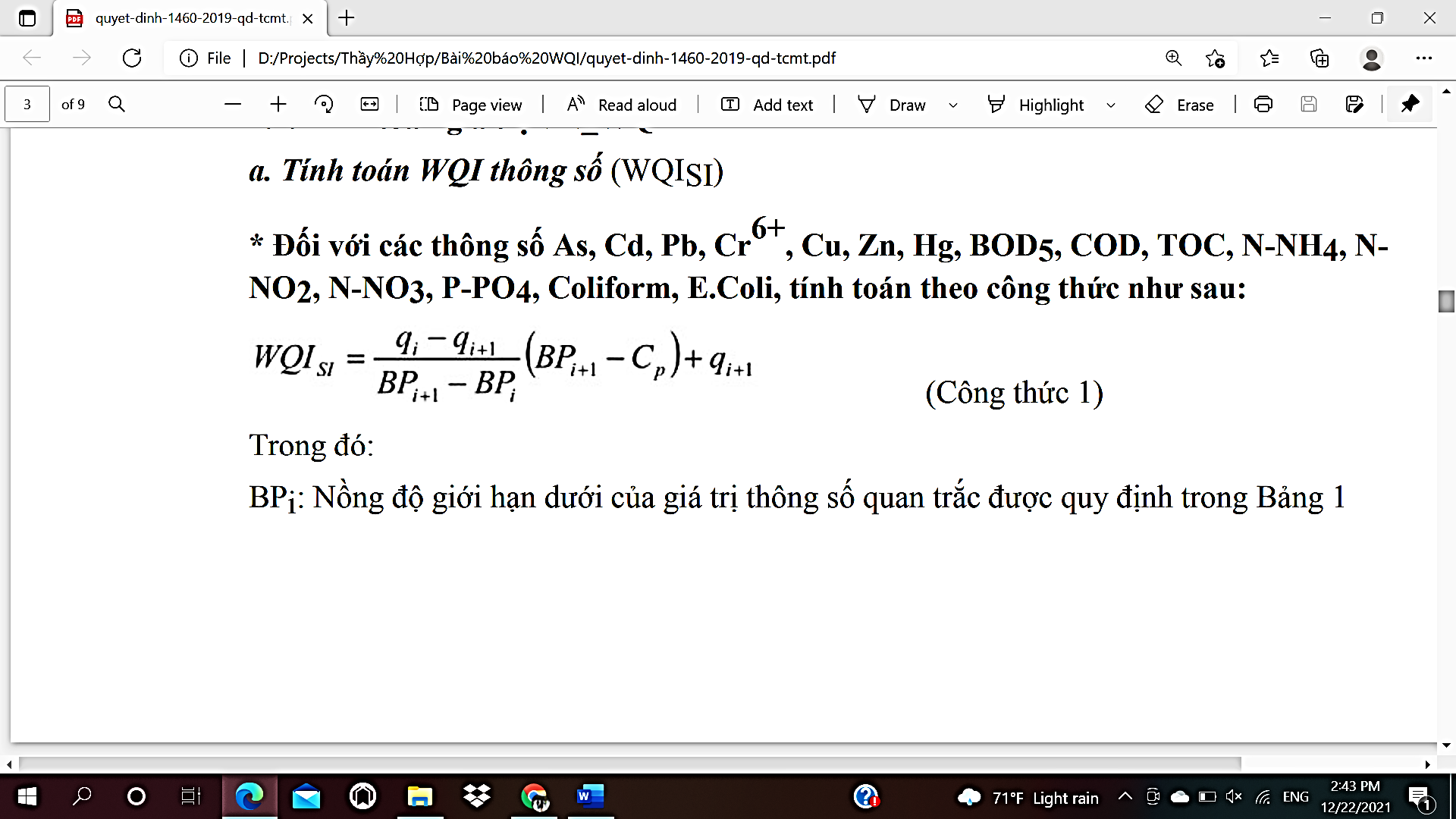
 (Eq.1)

In which:

BP_i_ : Lower limit concentration of the selected parameter specified in **Table 1** corresponding to level i;

BP_i+1_: Upper limit concentration of the selected parameter specified in **Table 1** corresponding to level i+1;

q_i_: WQI value at level i given in **Table 1** corresponding to BP_i_ value;

q_i+1_: WQI value at level i+1 given in **Table 1** corresponding to BPi+1 value

C_p_: The value of the monitored parameter used for the WQI_SI_ calculation.

**Table 1.** The specified q_i_, BP_i_ values for the selected parameters of groups IV and V^(*)^

| i | qi | BP_i_ value for each selected parameter | | | | | | | | |
| --- | --- | --- | --- | --- | --- | --- | --- | --- | --- | --- |
|  |  | BOD5 | COD | TOC | N-NH4 | N-NO3 | N-NO2 | P-PO4 | Total coliform | E.coli |
|  |  | mg/L | | | | | | | MPN/100 mL | |
| 1 | 100 | ≤ 4 | ≤ 10 | ≤ 4 | < 0.3 | ≤ 2 | ≤ 0.05 | ≤ 0.1 | ≤ 2500 | ≤ 20 |
| 2 | 75 | 6 | 15 | 6 | 0.3 | 5 |  | 0.2 | 5000 | 50 |
| 3 | 50 | 15 | 30 | 15 | 0.6 | 10 |  | 0.3 | 7500 | 100 |
| 4 | 25 | 25 | 50 | 25 | 0.9 | 15 |  | 0.5 | 10000 | 200 |
| 5 | 10 | ≥ 50 | ≥ 150 | ≥ 50 | ≥ 5 | ≥1 5 | ≥ 0.05 | ≥ 4 | ≥ 10000 | ≥ 200 |

**Table 2.** The specified q_i_, BP_i_ values for the selected heavy metals (group III)^(*)^

| i | qi | BP_i_ value for each selected parameter | | | | | | |
| --- | --- | --- | --- | --- | --- | --- | --- | --- |
|  |  | As^III,V^ | Cd^II^ | Pb^II^ | Cr^VI^ | Cu^II^ | Zn^II^ | Hg^II^ |
|  |  | mg/L | | | | | | |
| 1 | 100 | ≤ 0.01 | < 0.005 | < 0.02 | ≤ 0.01 | ≤ 0.1 | ≤ 0.5 | < 0.001 |
| 2 | 75 | 0.02 | 0.005 | 0.02 | 0.02 | 0.2 | 1.0 | 0.001 |
| 3 | 50 | 0.05 | 0.008 | 0.04 | 0.04 | 0.5 | 1.5 | 0.0015 |
| 4 | 25 | 0.1 | 0.01 | 0.05 | 0.05 | 1.0 | 2.0 | 0.002 |
| 5 | 10 | > 0.1 | ≥ 0.1 | ≥ 0.5 | ≥ 0.1 | ≥ 2 | ≥ 3 | ≥ 0.01 |

^(*)^ If the C_p_ value for a parameter equals to BP_i_ specified in the table, the WQI_SI_ for the parameter

is accepted to equal to the respective q_i_ value.

- WQI_SI_ calculation for parameter DO, based on DO% saturation value
- Step 1: calculate DO% saturation value (DO_saturation_)

DO_saturation_ = 14.652 – 0.41022T + 0.0079910T^2^ – 0.000077774T^3^

In which,

T: water temperature at the time of monitoring, ^o^C

DO% _saturation_ = (DO_dissolved_ / DO_saturation_) × 100

DO_dissolved_: the DO value measured at the time of monitoring, mg/L

- Step 2: Calculate WQI_SI_


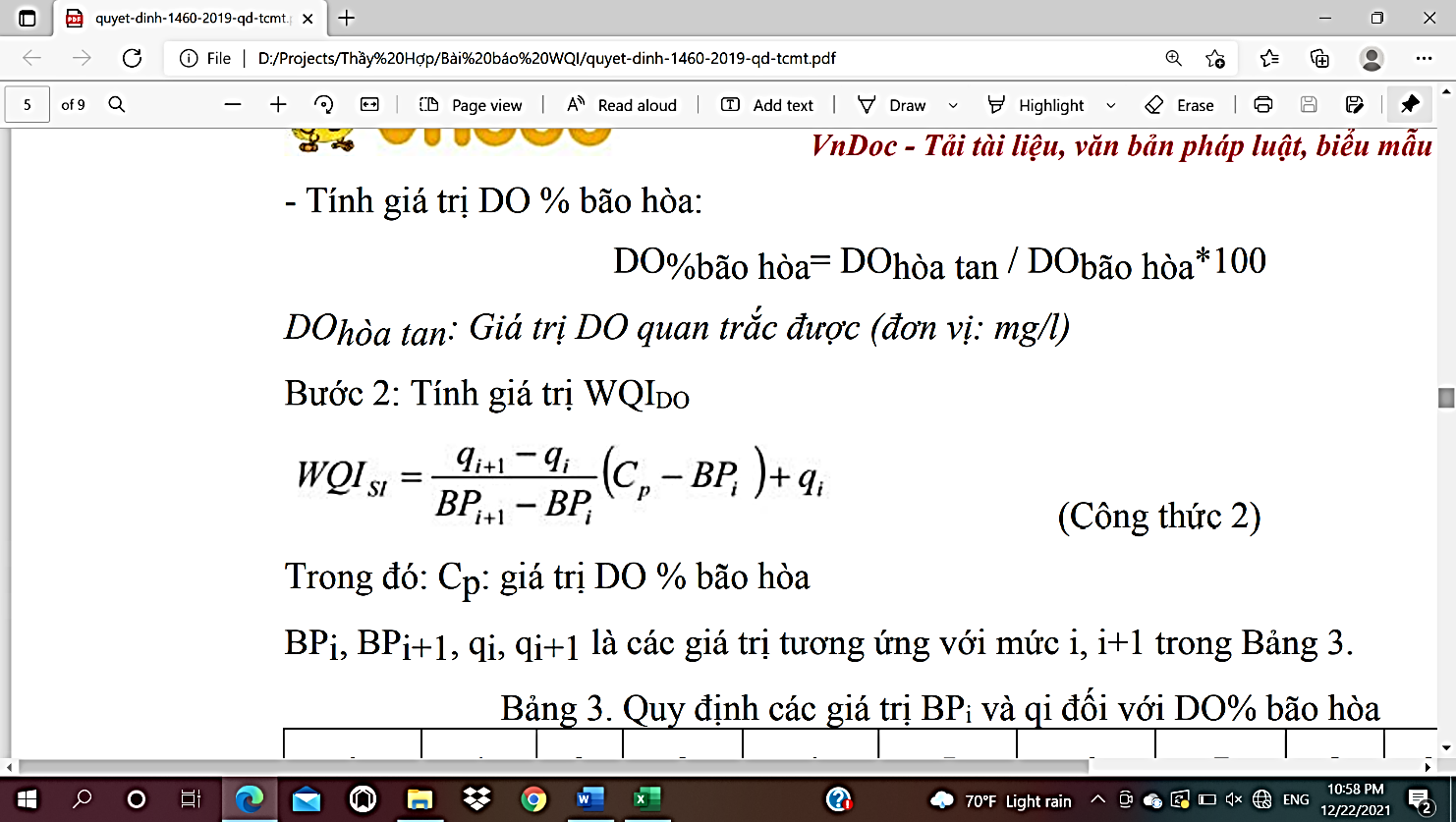


(Eq.2)

In which:

C_p_: DO% saturation value

BP_i_ , BP_i+1_, q_i_ , q_i+1_: the accordant values in **Table 3**

**Table 3.** The specified q_i_, BP_i_ values for DO% saturation value

| i | 1 | 2 | 3 | 4 | 5 | 6 | 7 | 8 | 9 | 10 |
| --- | --- | --- | --- | --- | --- | --- | --- | --- | --- | --- |
| BP_i_ | < 20 | 20 | 50 | 75 | 88 | 112 | 125 | 150 | 200 | > 200 |
| q_i_ | 10 | 25 | 50 | 75 | 100 | 100 | 75 | 50 | 25 | 10 |

If DO% saturation value < 20 or DO% saturation value > 200, then WQI_SI_ = 10.

If 20 < DO% saturation value < 88, then WQI_SI_ is calculated according to **Eq.2** and **Table 3**.

If 88 ≤ DO% saturation value ≤ 112, then WQI_SI_ = 100.

If 112 < DO % saturation value < 200, then WQI_SI_ is calculated according to **Eq.1** and **Table 3**.

- WQI_SI_ calculation for parameter pH:

**Table 4.** The specified q_i_, BP_i_ values for parameter pH

| i | 1 | 2 | 3 | 4 | 5 | 6 |
| --- | --- | --- | --- | --- | --- | --- |
| BP_i_ | > 5.5 | 5.5 | 6 | 8.5 | 9 | > 9 |
| q_i_ | 10 | 50 | 100 | 100 | 50 | 10 |

If pH < 5.5 or pH > 9, then WQI_SI_ = 10.

If 5.5 < pH < 6, then the WQI_SI_ is calculated according to **Eq.2** and **Table 4**.

If 6 ≤ pH ≤ 8.5, then the WQI_SI_ is 100.

If 8.5 < pH < 9, then WQI_SI_ is calculated according to **Eq.1** and **Table 4**.

- Calculation for the parameters in **Group II**: Aldrin, BHC, Dieldrin, DDTs, Heptachlor and Heptachlorepoxide

| Parameter | Measured concentration (µg/L) | WQI_SI_ |
| --- | --- | --- |
| Aldrin | ≤ 0.01 | 100 |
|  | > 0.1 | 10 |
| Benzene hexachloride (BHC) | ≤ 0.02 | 100 |
|  | > 0.02 | 10 |
| Dieldrin | ≤ 0.1 | 100 |
|  | > 0.1 | 10 |
| DDTs | ≤ 1.0 | 100 |
|  | > 1.0 | 10 |
| Heptachlor and Heptachlorepoxide | ≤ 0.2 | 100 |
|  | > 0.2 | 10 |

1. **Calculation of final WQI**


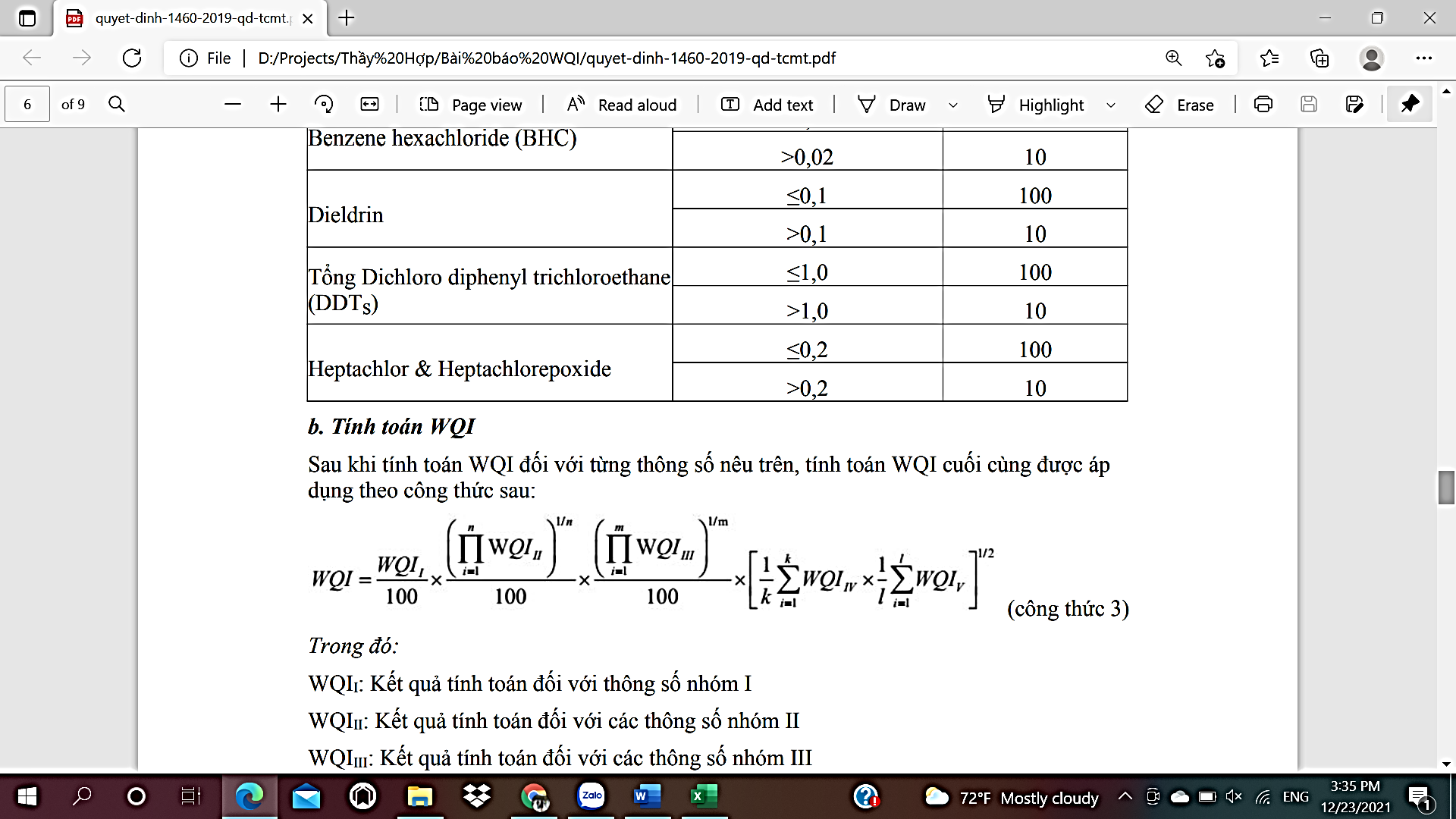


(Eq.3)

In which:

WQ_I_: WQI_SI_ calculation results for the parameters in Group I

WQ_II_: WQI_SI_ calculation results for the parameters in Group II

WQ_III_: WQI_SI_ calculation results for the parameters in Group III

WQI_IV_: WQI_SI_ calculation results for the parameters in Group IV

WQI_V_: WQI_SI_ calculation results for parameters in Group V

The final WQI is rounded up to be an integer.

1. **WQI classification**

| WQI range | Water quality | Color | RBG color codes |
| --- | --- | --- | --- |
| 91 – 100 | Excellent | Blue | 51;51;255 |
| 76 – 90 | Good | Green | 0;228;0 |
| 51 – 75 | Moderate | Yellow | 255;255;0 |
| 26 – 50 | Poor | Orange | 255;126;0 |
| 10 – 25 | Very poor | Red | 255;0;0 |
| < 10 | Highly polluted | Brown | 126;0;35 |
